# Intricate relationship between obstructive sleep apnea and dementia in older adults

**DOI:** 10.1007/s11357-023-00958-4

**Published:** 2023-10-09

**Authors:** Erica Ercolano, Leonardo Bencivenga, Maria Emiliana Palaia, Giovanni Carbone, Francesco Scognamiglio, Giuseppe Rengo, Grazia Daniela Femminella

**Affiliations:** 1 Department of Translational Medical Sciences, University of Naples “Federico II”, Via Pansini, 5, Naples, Italy; 2 Istituti Clinici Scientifici ICS Maugeri - S.P.A. - Istituti Di Ricovero E Cura a Carattere Scientifico (IRCCS) Istituto Scientifico Di Telese Terme, Telese, Italy; 3 Department of Brain Sciences, Imperial College London, London, UK

**Keywords:** Alzheimer’s disease, Obstructive sleep apnea, Biomarkers, Hypoxia

## Abstract

Numerous evidence reports direct correlation between cognitive impairment, Alzheimer’s disease and sleep disorders, in particular obstructive sleep apnea. Both obstructive sleep apnea and Alzheimer’s disease are highly prevalent conditions whose incidence increases with age. Several studies demonstrate how sleep-disordered breathing may lead to poor cognition, even though the underlying mechanisms of this association remain partially unclear. According to the most recent studies, obstructive sleep apnea may be considered a modifiable risk factor for cognitive dysfunction. In the present review, the authors aim to integrate recent research examining obstructive sleep apnea and Alzheimer’s disease biomarkers, also focusing on the mechanisms that support this correlation, including but not limited to the role of hypoxia and cardiovascular risk. Moreover, the potential favourable effect of obstructive sleep apnea therapy on cognitive function is discussed, to evaluate the benefits deriving from appropriate treatment of sleep-disordered breathing on cognition.

## Introduction

Obstructive sleep apnea (OSA) and Alzheimer’s disease (AD) are chronic conditions that cause significant morbidity and mortality among affected patients, exerting an enormous impact on socioeconomic national systems [1]. Sleep disturbances are an increasingly prevalent disorder, especially in the elderly, affecting around 1 billion people worldwide [2]. OSA is a type of sleep-disordered breathing (SDB) characterized by episodes of upper airway closure during sleep, resulting in impaired gas exchange with cycles of hypoxia/hypercapnia/reoxygenation, increased intrathoracic pressure swing, haemodynamic disruptions, chronic intermittent hypoxia (CHI) and sleep loss/fragmentation. The prevalence of OSA increases with age, estimated at 30–50% in people over 65 years old [3].

OSA is not diagnosed solely based on clinical assessment; rather, objective testing must be performed for accurate evaluation. This often involves nocturnal polysomnography (PSG). The severity of OSA is determined by the apnea-hypopnea index (AHI), which quantifies the number of apnoeic or hypopnoeic episodes per hour. Typically, an apnea is defined by a 90% decrease in respiratory airflow for 10 or more seconds, while hypopnea is defined as decrease of more than 30% in nasal airflow with a drop of at least 3% in oxygen saturation (AHI3a). Classification is based on the number of events per hour: mild OSA 4–15 events/hour, moderate OSA 15–29 events/hour and severe OSA more than 30 events/hour. OSA can be asymptomatic or can have an important impact on the quality of life, presenting with a wide range of clinical symptoms, such as snoring, unrefreshing sleep, excessive daytime sleepiness (EDS, measured with the Epworth sleepiness scale), cognitive impairment, depression and functional decline. Furthermore, Andrade et al. underlined how OSA often occurs in the context of several comorbidities like diabetes, dyslipidaemia, hypertension and obesity. Notably, it has been proposed that OSA should be included in the metabolic syndrome, leading to the term “syndrome Z” [4].

In 2020, an estimated 57 million people were living with dementia worldwide. According to the recent Global Burden of Disease (GBD) study [5], this estimate is expected to triple to 153 million in 2050, with the greatest increase in low- and middle-income countries. The 2020 report of the “Lancet Commission on dementia prevention, intervention, and care” highlighted 12 modifiable risk factors for dementia (low education, hypertension, hearing impairment, smoking, midlife obesity, depression, physical inactivity, diabetes, social isolation, excessive alcohol consumption, traumatic head injury and air pollution). Collectively, these factors account for approximately 40% of worldwide dementias. Public health interventions aimed at addressing these factors may theoretically prevent or at least delay dementia onset, reducing its prevalence [6].

Alzheimer’s disease (AD) is the most common cause of dementia. It is a neurodegenerative disorder affecting older adults, characterized by progressive decline in memory and cognitive functions, due to atrophy, degeneration, and progressive loss of neurons [7]. While familial early-onset AD is associated with genetic mutations [8, 9], the aetiology of the sporadic late-onset AD is largely unknown. The typical lesions are senile plaques determined by the aggregation of extracellular amyloid-β (Aβ) and neurofibrillary tangles (NFTs) of abnormally intracellular hyperphosphorylated tau protein [10, 11].

Mild cognitive impairment (MCI) describes the presence of cognitive decline with no or mild functional impact; MCI is an intermediate phase between normal cognitive ageing and overt dementia, to which this condition may evolve over time [12]. However, in some individuals, MCI reverts to normal cognition or remains stable; therefore, it is of extreme importance to identify which individuals with MCI are more likely to develop AD. The prevalence of MCI increases with age, reaching 10% in those aged 70–79 years and 25% among adults aged 80–89 years [13]. More recent study suggests that incidence may also be higher [14].

Based on the existing literature, the aim of this paper is to investigate whether there is an association between OSA and dementia, and to provide evidence suggesting the positive effect of OSA treatment on reducing risk of AD, in order to eventually apply disease-modifying therapy.

## Current evidence

There have been almost four decades of research looking for correlations between sleep disturbances and dementia, with first evidence that goes back to 1983 [15]. The relationship between sleep disturbance and dementia has been studied for decades [15], and it has been clarified over time thanks to increasing evidence on larger population and technological developments, both in diagnostic and therapeutic sections. Sharma and collaborators recently discovered an association between OSA and increased amyloid burden. However, the exact mechanism through which sleep apnea influences the longitudinal risk for Alzheimer’s disease has yet to be defined [1, 16]. Recent studies have directed their attention towards examining the cognitive profiles of patients with OSA [17, 18], and the pathophysiological continuum between OSA and AD [19] emphasizing the identification of shared biomarkers. However, numerous questions still remain unanswered, given that multiple potential pathophysiological mechanisms have been suggested to elucidate this relationship.

Similar to other sleep disturbances, OSA is responsible for changes in cognitive functions regulated by sleep, such as memory consolidation [20]. Neurodegenerative modifications have been observed in patients with intermittent hypoxia, sleep fragmentation and intrathoracic pressure swings, attributable to OSA. In particular, hypoxia promotes increased production and accumulation of Aβ42 as reported in several cerebral ischemia studies [21–23], while chronic intermittent hypoxia, hypercapnia and hypertension are responsible for neuronal damage to axons [24], white matter [25], and reduced diffusion tensor imaging (DTI)-based mean diffusivity (MD) in multiple brain regions [26].

White matter is extensively affected in OSA patients [27]; the alterations affect fibres of the anterior corpus callosum, anterior and posterior cingulate cortex and cingulum bundle, right column of the fornix, portions of the frontal, ventral prefrontal, parietal and insular cortices, bilateral internal capsule, left cerebral peduncle, middle cerebellar peduncle and corticospinal tract and deep cerebellar nuclei [28]. Studies have also shown focal grey matter loss (including bilateral caudate nuclei and the pre-frontal circuit) in OSA patients compared to controls [29]. These findings may partially contribute to explain the impairment in executive functions in OSA patients who develop dementia. In other studies, a reduction in grey matter volume was observed in the right middle temporal gyrus and the left cerebellum, in a large sample of OSA patients; these neural deficits could lead to impaired motor processing, divided attention tasks and working memory [30]. The presence of tissue damage in different regions may explain the different cognitive domains involved.

Cognitive decline that progressively turns into AD could also manifests as consequence of pathophysiological effects of hypoxia, such as precipitating hypertension [31, 32], cerebral chronic hypoperfusion [33, 34], impaired glucose metabolism [35–37] and adverse cardiovascular and metabolic consequences [38]. Therefore, it is clear that older patients with OSA and affected cardiovascular system might be at higher risk of AD than those without OSA-related vascular symptoms. Also inflammation [39, 40] and oxidative stress [41, 42] have been reported to contribute to neurocognitive impairment in OSA.

Alterations in the cerebrospinal fluid-interstitial fluid (CSF-ISF) exchange, promoted by the glymphatic system, constitute another possible cause of increased AD risk in OSA. Indeed, glymphatic flow of metabolites from ISF to CSF is hindered by intrathoracic pressure swings resulting from respiratory efforts against a closed airway; even if an increased pressure may still lead to elevated flow, it contributes to dysfunction [43–45]. Venous overload which may be elevated in OSA can determine a reduction in the clearance of subarachnoid CSF directly into dural lymphatic channels. Lastly, cerebral oedema may happen as consequence to intermittent hypoxia.

Recently, another study has provided further evidence of the association between OSA and higher brain amyloid burden [46]. In their research, Jackson and colleagues included 34 people with untreated OSA (mean age 57.5 ± 4.1) and 12 healthy controls (mean age 58.5 ± 4.2); both groups underwent clinical polysomnogram and a C-PiB positron emission tomography (PET) scan to quantify amyloid burden. Amyloid burden was elevated in the OSA group compared to controls and was significantly higher in those with severe OSA. Higher amyloid burden was associated with a higher non-REM apnea–hypopnea index, poorer sleep efficiency and less time spent in stage N3 sleep (deepest phase of sleep), when controlling for age. The study was the first to compare brain Aβ burden between individuals with polysomnographically-diagnosed untreated OSA and age-/gender-matched healthy controls and the authors concluded that severe OSA is associated with a modest elevation of brain amyloid [46].

Furthermore, people with OSA may have reduced hippocampal volume. Owen found inverse correlation with OSA severity, and although OSA may be a risk factor for AD, the hippocampus and brainstems of clinically verified OSA cases have not yet been examined for NFTs and Aβ plaques [47].

## Mechanisms

Emamian et al. [48] found that AD patients have a fivefold increased risk of presenting OSA compared to same-age controls, and in turn, OSA may worsen existing AD. Moreover, these data suggest that around half of patients with AD might have experienced OSA after their initial diagnosis.

Yaffe and colleagues [49] examined the association between sleep-disordered breathing detected with polysomnography, and subsequent diagnoses of MCI and dementia to look for evidence that sleep-disordered breathing precedes cognitive impairment and assess possible causal mechanisms (hypoxia, sleep fragmentation or sleep duration) to explain this association. They discovered that among older women, sleep-disordered breathing was associated with an increased risk of developing cognitive impairment during following 5 years.

In a recent study on cognitively normal adults, OSA was associated with increased amyloid burden over a 2-year follow-up, supporting the idea that it constitutes an important risk factor for developing AD [1] through several mechanisms that impair the structural integrity of the brain. Features such as sleep fragmentation, intermittent hypoxia and intrathoracic pressure swings are likely candidate etiological mechanisms.

**Fig. 1:**
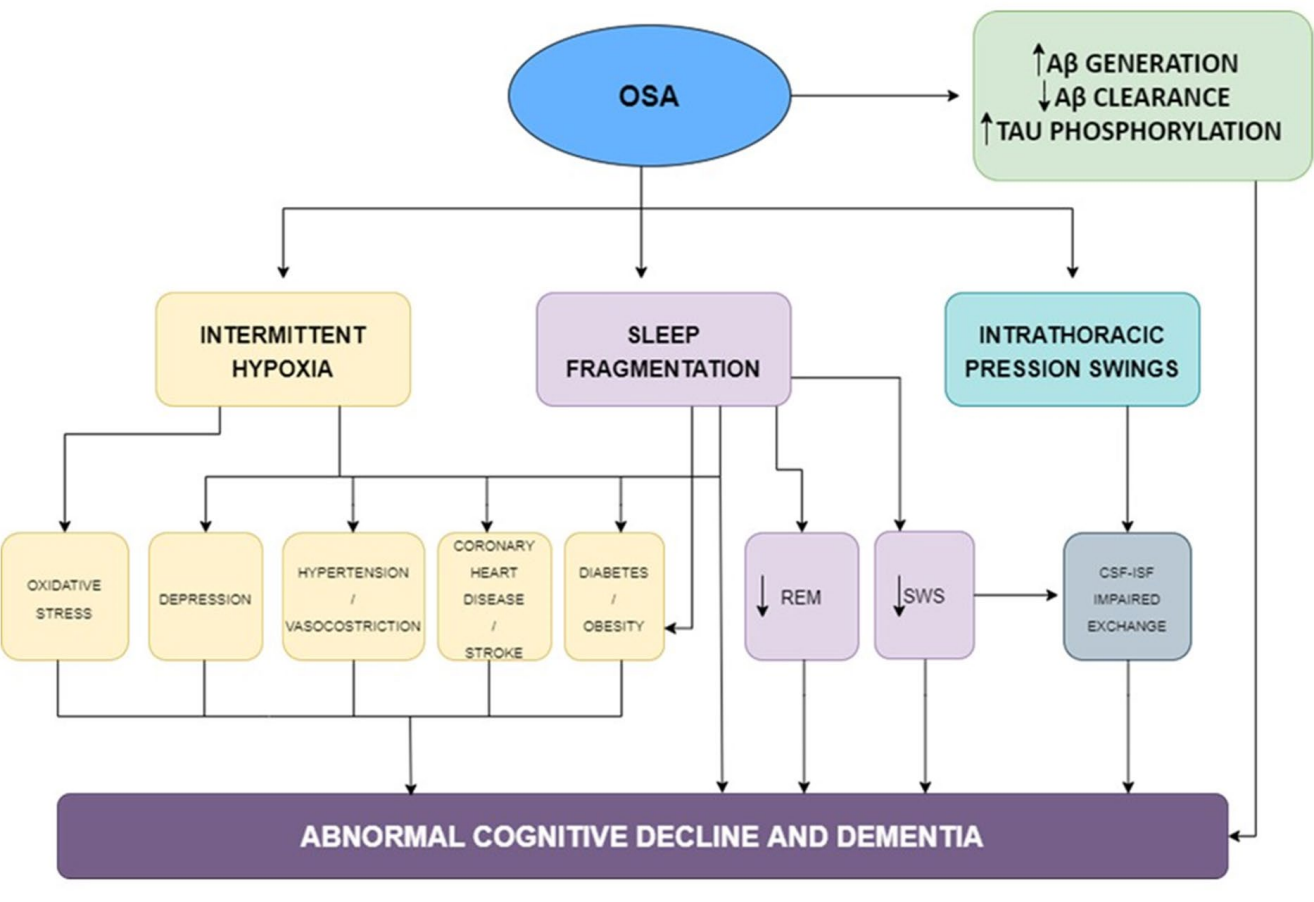
Mechanisms linking OSA and dementia. Schematic representation of the possible mechanisms linking OSA to dementia. OSA leads to intermittent hypoxia and changes in sleep macro- and microarchitecture. Intermittent hypoxemia may cause, oxidative stress, inflammation, hypertension, blood–brain barrier dysfunctions, and metabolic disturbances such as diabetes; all potentially contributing to the development of AD. Furthermore, intrathoracic pressure swings may disrupt CSF-ISF exchange integrity and lead to AD neuropathology accumulation. Adapted from Bubu et al. [101] (license number 5630961337227)

Figure 1 shows the possible mechanisms linking OSA to the risk of dementia.

### OSA and intermittent hypoxia

The oxygen desaturation index (ODI) is commonly used to measure intermittent hypoxia and quantifies the number of dips in oxygen saturation level by 3% or more per hour of sleep, while the time in apnea or hypopnea estimates the proportion of the sleep period during which the respiration consists of apneas and hypopneas [49, 50]. Unlike the apnea-hypopnea index that simply counts apneas and hypopneas per hour of sleep, the percentage of time in apnea or hypopnea reflects both the frequency and duration of breathing disturbances and may better indicate sleep-related gas exchange abnormalities. Studies suggest intermittent hypoxia, rather than continuous hypoxia, to be associated with greater risk of oxidative stress and adverse outcomes such as metabolic dysfunction, including dyslipidemia and insulin resistance [49, 51].

Apneas and hypopneas may provoke adaptive (e.g. ischemic preconditioning) but also maladaptive and potentially harmful responses (e.g. oxidative stress, inflammation, hypertension, dysautonomia, impaired glucose tolerance and blood–brain barrier dysfunction) that may in turn damage cerebral tissues, and make neurons more susceptible to cellular death [40].

In particular, numerous studies have established that intermittent hypoxia (IH) is implicated in the activation and progression of inflammation in OSAS patients through different mechanisms. Studies suggest intermittent hypoxia, rather than continuous hypoxia, to be associated with greater risk of oxidative stress and adverse outcomes such as metabolic dysfunction, including dyslipidemia and insulin resistance [52]. The severity of nocturnal intermittent hypoxemia has been associated with impairment of insulin sensitivity and insulin secretion by pancreatic beta-cells [53]. Leptin also is increased in OSAS patients perhaps because IH is a potent stimulator of leptin. The dysregulation of leptin levels promotes oxidative stress and increased production of IL-6 and TNF-α [52, 53].

Studies of cerebral ischemia suggest that both acute and intermittent hypoxia may also promote Aβ accumulation, since hypoxia in OSAS reduces neuronal activity. In different models, hypoxia is associated with increased Aβ expression and reduced degradation, which might lead to Aβ accumulation [54].

### OSA and intrathoracic swings

During episodes of OSA, the attempts to breathe against a closed or partially obstructed airway lead to significant swings in intrathoracic pressure. These swings occur as the individual exerts extra effort to inhale despite the obstruction. When the airway opens, there is a sudden release of pressure, and when it closes again, pressure increases once more. Various pathophysiological mechanisms have been hypothesized to underlie the relationship between OSA and intrathoracic pressure swings [55].

CSF dynamic in humans is still poorly understood; however, its flux is involved in the pathophysiology of neurodegenerative diseases and cognitive impairment, usually after traumatic brain injury, mainly due to the accumulation of cellular metabolic waste. Dreha-Kulaczewski and collaborators [44] identified forced inspiration as a main driving force of CSF flow in the human brain by use of real-time phase-contrast MRI. They analyzed directions, velocities and volumes of human CSF flow within the brain aqueduct as part of the internal ventricular system and in the spinal canal during respiratory cycles. A consistent upward CSF movement toward the brain in response to forced inspiration was observed in all participants. The authors hypothesize that intrathoracic pressure swings from respiratory efforts against a closed airway would impede the glymphatic flow of metabolites from interstitial fluid to the CSF. The critical role of CSF comprises indeed the maintenance of immunologic and biochemical homeostasis and the supply of essential microenvironment for brain health and function. The choroid plexus (CP) adhering to the walls of the ventricular system controls the blood–CSF barrier and tightly regulates CSF composition [56]. Multiple bidirectional transport systems within the CP facilitate the secretion of essential ions and molecules into and favour the clearance of unnecessary components out of the CSF [57]. Furthermore, increased venous pressure that is typically elevated in OSA may lead to a reduction in the clearance of subarachnoid CSF directly into dural lymphatic channels, although this pathway is still not clear, as a minor dural lymphatic clearance has been observed in rabbits [58] and rats [59].

Indeed, the glymphatic system which promotes solute exchange between CSF and the interstitial fluid is affected by repetitive fluctuations in intrathoracic and intracranial pressures in OSAS. These might impede the flow of metabolites from the interstitial space to the CSF, including Aβ, which might lead to its accumulation [54]. A more plausible mechanism may be related to cerebral oedema secondary to intermittent hypoxia that reduces interstitial space expansion hence not promoting CSF homeostasis.

### OSA and sleep disturbances

Sleep is broken down into 4 phases: N1, N2, N3 and R. Stages N1 to N3 are considered non-rapid eye movement sleep (NREM). N3 is the third and deepest stage of sleep, also known as slow-wave sleep (SWS), characterized by slower frequency with high amplitude signals known as delta waves. As people get older, they tend to spend less time in this slow delta wave sleep and more time in N2 sleep [60]. Studies in cognitively normal elderly show how reduced or fragmented SWS is associated with increased Aβ42 in CSF, suggesting that disturbed sleep might drive an increase in soluble brain Aβ levels prior to amyloid deposition [61].

Insufficient or poor-quality sleep may provide a lot of deleterious effects, since sleep wake rhythm is involved in immune system modulation, weight management, glucose metabolism, cardiovascular and cerebrovascular health, cognition, work productivity and psychological health [62, 63]. Xie and colleagues studied how sleep may carry out his critical effects [64], since sleep subserves the important function of clearing multiple potentially neurotoxic central nervous system degradation products. In their study, they showed how natural sleep is associated with a 60% increase in the interstitial space, resulting in a higher convective exchange of cerebrospinal fluid with interstitial fluid, while convective fluxes of interstitial fluid increased the rate of Aβ clearance during sleep. Furthermore, REM phase seems to be implicated in sleep-related synaptic consolidation processes [65–67], and the higher rate of apneas during sleep in OSA patients could lead to a disruption of this memory promoting processes. Higher levels of cortisol in OSA patients are indicative of hippocampal atrophy and memory impairment, associated with cognitive decline, as reported by Lupien [68].

The most common symptoms of sleep apnea include excessive daytime sleepiness, fatigue, restless sleep and morning headache. All these symptoms may be related to changes in sleep architecture, including sleep fragmentation, due to the repeated occurrence of end-apneic arousal throughout the night, and decrease in slow-wave sleep (SWs) and REM sleep [69]. More specifically, sleep fragmentation and reduced SWS can increase Aβ deposition [70]. Early studies suggest that the degree of sleep fragmentation results in deficits of attention and memory, while the severity of hypoxemia influences motor function, vigilance and executive function.

During OSA, the airway obstruction leads to reduced oxygen levels (hypoxemia) and elevated carbon dioxide levels (hypercapnia). These factors trigger an enhanced respiratory response. Nonetheless, this heightened urge to breathe is often futile in the absence of a natural airway reopening. Consequently, the apnea or hypopnea events persist until the patient awakens from sleep, ultimately resolving the airway obstruction [55].

### OSA and cardiovascular manifestations

OSA is also a risk factor for development of cardiovascular disorders, and vice versa [71]. The pathophysiological mechanisms that cause increased vascular risk are still matter of debate. Several mechanisms have been proposed to explain the increased cardiovascular risk in OSA patients, including sympathetic system activation, oxidative stress [72], local and systemic inflammation, endothelial dysfunction, hypercoagulability [73] and metabolic dysregulation. OSA likely contributes to the development or progression of heart failure and heart failure might also contribute to development of OSA. During OSA, negative inspiratory intrathoracic pressure generated against the occluded upper ways increases left ventricular transmural pressure, and therefore the afterload. During OSA, increased venous return right ventricular pre-load is observed, associated with increased right ventricular afterload due to OSA-induced hypoxic pulmonary vasoconstriction. This causes right ventricular distension and impaired left ventricular filling. The combination of increased left ventricular afterload and reduced preload reduces stroke volume and cardiac output [74].

**Fig. 2:**
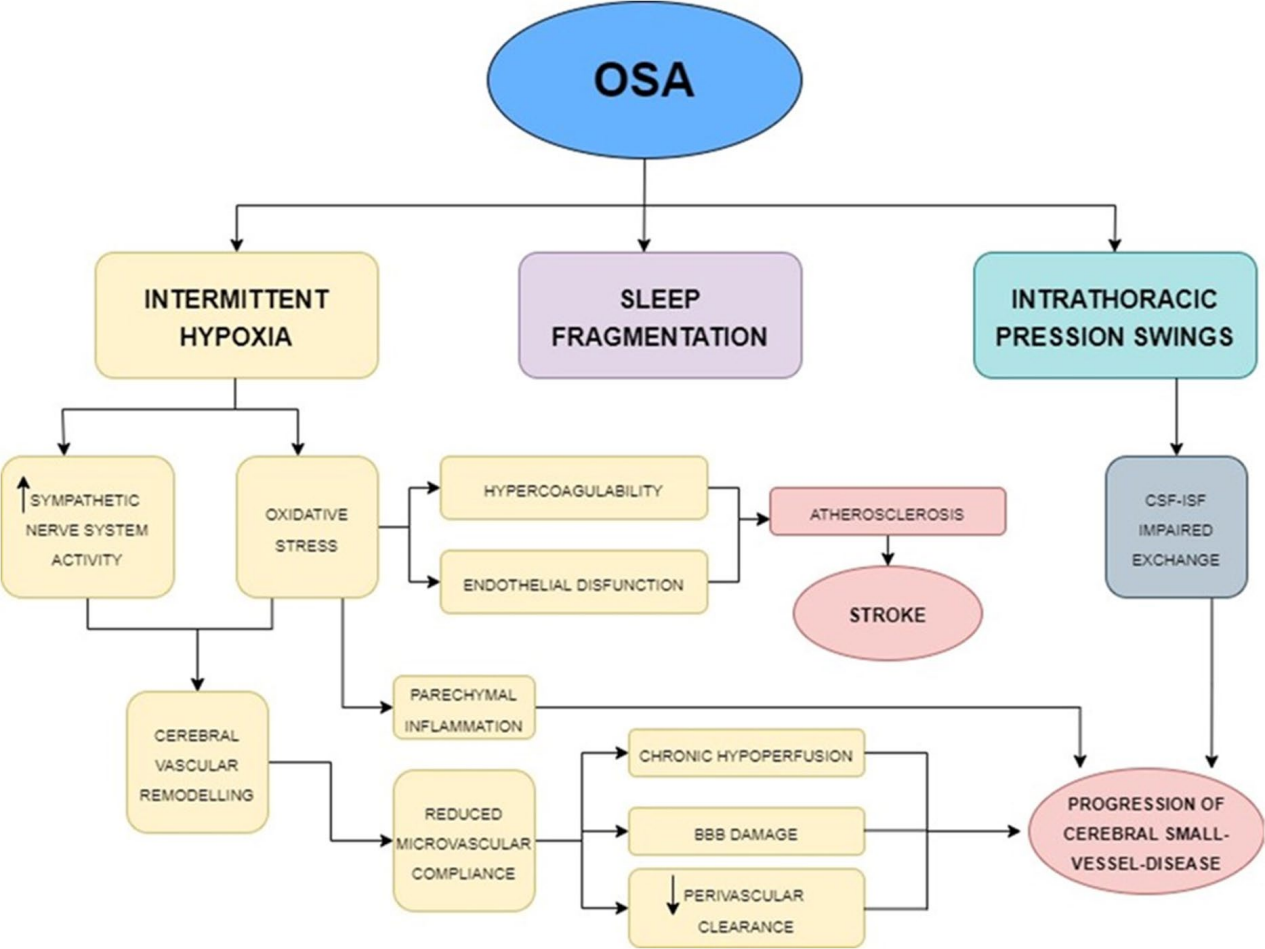
Mechanisms underlying cerebrovascular changes in OSA. Pathophysiologic mechanisms dependent on the cerebral vascular remodeling process. All the systemic changes caused by OAS, combined with the altered sleep macro- and microarchitecture, may lead to small-vessel disease, microinfarcts, strokes, reduced synaptic plasticity, decreased cognitive functioning, changes in brain white and gray matter, changes in cerebral networks, and abnormal levels of Alzheimer’s disease biomarkers. Adapted from Bubu et al. [101] (license number 5630961337227)

Studies have shown that severe OSA increases the risk of cerebral small-vessel disease [75] and stroke [76, 77]. Figure 2 shows the possible link between OSA and cerebrovascular disease. Accordingly, OSA and small-vessel disease (SVD) share common risk factors; moreover, OSA also influences the pathogenesis of SVD by multiple mechanisms [78]. OSA is associated with the progression of SVD through endothelial dysfunction and decreased vascular compliance [78]. The endothelial dysfunction in OSA may be related to the consequent generation of reactive oxygen species (ROS) and pro-inflammatory molecules, resulting in microvascular damage in OSA patients [79]. In particular, the intermittent hypoxia occurring during OSA can activate the sympathetic nervous system and induce oxidative stress with parenchymal inflammation [80]. Also, altered intrathoracic pressure might impair the activation of cerebral waste clearance system during sleep, which is a crucial mechanism to maintain brain homeostasis through the circulation of brain metabolites from the interstitial fluid into the CSF through the glymphatic system, leading to increases in Aβ accumulation [54]. The inflammatory cascade may also result in the disruption of the CSF integrity and the glial and neural cell damage [81, 82]. Moreover, Buratti et al. [83] established that intima tunica and media thickness in AD patients carotid artery associate with severity of OSA.

Furthermore, OSA may be also linked to a higher risk of stroke by its effect on vascular risk factor, as aforementioned [84]. Increased negative intrathoracic pressure swing during apnoeic phases of OSA increases cardiac vagal output that promotes atrial fibrillation [85], which is a major risk factor for stroke, particularly cardioembolic stroke.

Several studies demonstrate that patients with OSA show elevated fibrinogen levels and increased platelet aggregation that are reversed with continuous positive airway pressure treatment (CPAP). There is a direct association between the severity of OSA in patients with stroke and the plasma level of fibrinogen [86]. Hypercoagulability has been studied as an underlying factor [87], and several studies suggest that patients with moderate to severe OSA have elevated blood coagulability markers compared with healthy individuals, which may contribute to the occurrence of cardiovascular complications, such as stroke [88].

## Therapy

Specific interest in OSA stems from speculation that its effective treatment can slow, reverse or even prevent the onset of dementia, particularly for patients starting treatment after the age of 65 [89]. Indeed, since OSA can be efficiently treated with continuous positive airway pressure (CPAP), it is interesting in the context of its association with dementia. Several clinical studies, exploring the effect of CPAP treatment on cognition and AD, reinforce the suspected link between OSA and AD.

CPAP approach has been shown to slow or even improve cognitive impairment [90]. Furthermore, a recent investigation of individuals with sleep apnea indicated that treatment with CPAP improved cognitive scores, and also increased grey matter volume in the hippocampal and frontal regions [91]. Castronovo and colleagues [91], in their prospective clinical study, demonstrate how a 12-month CPAP treatment can significantly improve memory, attention and executive-functioning and even determine almost complete reversal of white matter abnormalities.

Early studies show how an adequate CPAP treatment can restore sleep macrostructure, in particular slow waves (SWs) and REM duration increases while sleep latencies decreases [92, 93]. OSA treatment, such as CPAP, revealed effective in reducing hypopneic and apneic events. In a randomized study of 33 patients with OSA aged 71.3 ± 5.5 years, 3 months of CPAP improved short-term memory, working memory, selective attention and executive functions as well as functional connectivity in the right middle frontal gyrus [89]. A recent study on a non-dement cohort of elderly subjects, enrolled in the Alzheimer’s Disease Neuroimaging Initiative (ADNI) study, showed that CPAP treatment delayed the age of MCI onset by approximately 10 years (72 versus 82 years old) [90].

Liguori and collaborators [94] showed that 1 year of CPAP treatment normalized the CSF Aβ42 and t-tau/Aβ42 ratio levels as well as the cognitive complaints, suggesting that OSA might be a reversible risk factor for dementia. The relationship between OSA and metabolic changes is reinforced by studies of the benefits of CPAP. In fact, CPAP treatment improves glucose intolerance in the short term and long term [95, 96], and glycaemic control [97]. Given the high prevalence of SDB and cognitive impairment among older adults, several studies suggest the need to examine the prospective benefit of CPAP therapy for prevention or delay of MCI onset and cognitive decline [90].

Despite all the benefits, nightly CPAP use remains problematic for some patients; therefore, alternative treatment approaches have been adopted, such as oral pressure therapy, oral appliances (such as tongue retaining devices or mandibular advancement devices MADs), surgical treatment (adenotonsillectomy, nasal surgeries, palatal surgeries, tongue-based surgeries, multilevel surgeries), hypoglossal nerve stimulation, drug treatment or the combination of two or more of the aforementioned treatments [98]. For those patients who refuse the use of devices or surgery, positional therapy and weight loss/exercise should be considered part of a multimodal treatment approach [98].

Despite the many options available to treat OSA, none of them is as efficacious as CPAP [99]. For those patients who are CPAP intolerant, nasal expiratory positive airway pressure (nEPAP) may be a reasonable alternative with positive benefits (e.g. decreased AHI, improved oxygen saturation, increased quality of life, decreased snoring) and very few side effects (e.g. dry mouth, nasal discomfort); although adherence would still be a problem, it is better when compared to CPAP [100].

## Discussion

The relationship between OSA and cognitive impairment has garnered significant attention in recent years. While there is a growing body of evidence supporting this association, it is important to know the limitations of the current research. Most studies establish an association between OSA and cognitive impairment, but causality still remains challenging to confirm. It is unclear whether OSA directly leads to cognitive decline or if other factors, like common comorbidities, contribute to both conditions. Furthermore, many individuals with OSA also have other health conditions, such as obesity, hypertension or diabetes, which can independently impact cognitive function; untangling the specific contribution of OSA is difficult.

Previous narrative and systematic reviews have extensively summarized findings on the association between OSA and cognitive decline, either focusing on specific pathophysiological mechanisms, such as neuroinflammation, or reporting findings from clinical studies [101–103]. In this review, we aimed to highlight current evidence on the main mechanisms underlying the association between OSA and AD, including clinical and preclinical studies. Moreover, we report the main findings from studies on OSA treatment which might have an impact on cognitive functions. While the evidence linking OSA and cognitive impairment is growing, there are still several limitations that need to be clarified in future research. Overcoming these challenges will provide a clearer understanding of the nature of the relationship, its underlying mechanisms and potential strategies for intervention and treatment. Therefore, this review aims to give some potential areas for future studies to investigate the impact of OSA on cognition:

- Neuroimaging and brain connectivity: use of advanced neuroimaging techniques, such as functional MRI (fMRI) and diffusion tensor imaging (DTI), to investigate alterations in brain structure and connectivity associated with OSA-related cognitive impairment. This could help identify specific brain regions affected by OSA and understand the underlying neural mechanisms.
- Sleep architecture and cognitive domains: explore how specific sleep architecture disruptions in OSA are linked to impairments in different cognitive domains. Understanding these relationships could lead to targeted interventions to improve specific cognitive functions.
- Impact of sleep fragmentation: explore the effects of sleep fragmentation on cognitive function in OSA. This could help differentiate the contributions of IH and sleep disruption to cognitive impairment.
- Influence of comorbidities: investigate the role of comorbid conditions frequently associated with OSA in exacerbating or interacting with OSA-related cognitive impairment.
- Genetic factors: study the genetic factors that might predispose individuals with OSA to cognitive deficits. This could lead to the identification of biomarkers or genetic variants associated with increased vulnerability to OSA-related cognitive impairment.
- Interventional studies: design intervention studies that combine cognitive training/rehabilitation with OSA treatment, to determine which approach can have larger benefits for cognitive recovery.
- Population studies: conduct large-scale population studies to determine the prevalence of cognitive impairment in individuals with undiagnosed OSA.

By exploring these areas, researchers can further unravel the intricate relationship between OSA and cognitive impairment, paving the way for improved diagnostic strategies, targeted interventions and enhanced patient care.

## Conclusions

Growing evidence from recent studies supports an interdependent relationship between OSA and AD. The detrimental impact of OSA in cognition, particularly on executive function and attention, may contribute to a worse clinical presentation of AD. This is further supported by the observation that appropriate diagnosis and treatment of OSA show promising beneficial preventive effect in preclinical AD as well as in slowing cognitive declines in clinical AD. To comprehensively assess the impact of treatment for sleep-disordered breathing in elderly populations, additional trials with larger sample sizes, longer treatment periods and more diverse populations are required.
